# In Vitro Effect on Piglet Gut Microbiota and In Vivo Assessment of Newly Isolated Bacteriophages against F18 Enterotoxigenic *Escherichia coli* (ETEC)

**DOI:** 10.3390/v15051053

**Published:** 2023-04-25

**Authors:** Margaux Navez, Céline Antoine, Fanny Laforêt, Elizabeth Goya-Jorge, Caroline Douny, Marie-Louise Scippo, Marjorie Vermeersch, Jean-Noël Duprez, Georges Daube, Jacques Mainil, Bernard Taminiau, Véronique Delcenserie, Damien Thiry

**Affiliations:** 1 Laboratory of Bacteriology, Department of Infectious and Parasitic Diseases, FARAH and Faculty of Veterinary Medicine, University of Liege, 4000 Liege, Belgium; 2 Unit of Cardiovascular Sciences, Groupe Interdisciplinaire de Génoprotéomique Appliquée (GIGA), University of Liege, 4000 Liege, Belgium; 3 Laboratory of Food Quality Management, Food Science Department, FARAH and Faculty of Veterinary Medicine, University of Liège, 4000 Liege, Belgium; 4 Laboratory of Food Analysis, Department of Food Sciences, FARAH and Faculty of Veterinary Medicine, University of Liège, 4000 Liege, Belgium; 5 Center for Microscopy and Molecular Imaging, Electron Microscopy Laboratory, ULB, 6041 Gosselies, Belgium; 6 Laboratory of Food Microbiology, Fundamental and Applied Research for Animals & Health (FARAH), Department of Food Sciences, Faculty of Veterinary Medicine, University of Liege, 4000 Liege, Belgium

**Keywords:** bacteriophages, phage therapy, ETEC, *Galleria mellonella*, weaning piglets, microbiota, post-weaning diarrhea, SHIME

## Abstract

Enterotoxigenic *Escherichia coli* (ETEC) causing post-weaning diarrhea (PWD) in piglets have a detrimental impact on animal health and economy in pig production. ETEC strains can adhere to the host’s small intestinal epithelial cells using fimbriae such as F4 and F18. Phage therapy could represent an interesting alternative to antimicrobial resistance against ETEC infections. In this study, four bacteriophages, named vB_EcoS_ULIM2, vB_EcoM_ULIM3, vB_EcoM_ULIM8 and vB_EcoM_ULIM9, were isolated against an O8:F18 *E. coli* strain (A-I-210) and selected based on their host range. These phages were characterized in vitro, showing a lytic activity over a pH (4–10) and temperature (25–45 °C) range. According to genomic analysis, these bacteriophages belong to the *Caudoviricetes* class. No gene related to lysogeny was identified. The in vivo *Galleria mellonella* larvae model suggested the therapeutic potential of one selected phage, vB_EcoS_ULIM2, with a statistically significant increase in survival compared to non-treated larvae. To assess the effect of this phage on the piglet gut microbiota, vB_EcoS_ULIM2 was inoculated in a static model simulating the piglet intestinal microbial ecosystem for 72 h. This study shows that this phage replicates efficiently both in vitro and in vivo in a *Galleria mellonella* model and reveals the safety of the phage-based treatment on the piglet microbiota.

## 1. Introduction

The enterotoxigenic pathotype of *Escherichia coli*, shortly named ETEC, is considered as the most common bacterial cause of traveler’s and children’s diarrhea in developing countries. More than 157,000 fatal cases of human diarrhea are annually caused by ETEC according to the World Health Organization report [1]. ETEC can adhere to the small intestine epithelial cells via surface structures and secrete enterotoxins, which are the most important and effective virulence factors of the bacteria. Colonization factors (CF) are the second main virulence factors involved in ETEC diarrhea [2]. ETEC interact with the small intestinal epithelial cells through various plasmid-encoded colonization factor genes encoding fimbrial, non-fimbrial and fibrillar structures [3,4].

In piglets, ETEC infection often leads to post-weaning diarrhea (PWD) resulting in significant morbidity and mortality (up to 25%) a few days after weaning [5,6]. Moreover, according to research on the prevalence of *E. coli* virulence factors in Europe, 59.6% of the farms under investigation have been tested positive for ETEC in samples from diarrheal swine [7]. This disease also involves production losses, treatment costs, isolation costs, feed supplementation or other managemental care employed to treat the disease [8]. The weaning period is a source of intense stress due to many changes such as adaptation to a new environment, cohabitation with new pigs, dietary changes and histological variations in the small intestine. All these events weaken the immune system and lead to intestinal dysfunctions, facilitating a significant proliferation of *E. coli* and the development of PWD [9].

PWD is first diagnosed based on clinical signs and farm history, but strain isolation and PCR typing of fimbriae and enterotoxin genes are necessary for a final, conclusive diagnosis. The control of the infection starts with the management optimization of pig reproduction. Treatment, metaphylaxis and prevention of ETEC infection are generally covered with antibiotics such as amoxicillin/clavulanic acid, apramycin, gentamicin or also neomycin. One possible candidate for the treatment of PWD in case of antibiotic resistance is colistin sulfate. Studies have reported the emergence of colistin resistance, which may be due to modifications of LPS with the addition of positively charged groups or also due to the presence of a plasmid-mediated *mcr* resistance gene [10,11]. This massive use of antibiotics leads to antimicrobial resistance detected in ETEC strains globally. The impact on the swine industry is again accentuated by the increased mortality and costs associated with extended treatments [12].

Phage therapy has been reported to alleviate the severity of clinical signs induced by bacterial infections in animals [13,14,15]. This therapy could offer the pig industry a highly targeted therapeutic strategy. A study conducted by C. Y. Lee et al. aimed to investigate the effects of dietary phage supplementation against ETEC K88 as part of the porcine colibacillosis treatment [15]. This supplementation was associated with a reduction in intestinal and fecal *E. coli* scores that tended to decrease clinical signs. Furthermore, due to the low pH of some parts of the gastrointestinal tract, it is important to protect unstable phages from pH variations to avoid the inactivation of the viruses before they can reach their target in the distal small intestine. Several ways to protect the bacteriophage were studied, such as encapsulation or pre-administration of antacids [16].

The greater wax moth larva (or *Galleria mellonella* larvae) model has become a widely adopted insect model to study several infections including diseases caused by *E. coli*. The larvae can be reared at various temperatures (20–40 °C) and can tolerate prolonged incubation. The manipulation of these larvae is facilitated by their size, and survival studies can be easily performed by monitoring their activity and melanization [17]. Moreover, the *Galleria mellonella* larvae model is more ethically acceptable than other animal models [18] and has the main advantage of having an innate immune system quite similar to mammals [19]. Several publications have demonstrated the benefits of this model to assess phage therapy and its effectiveness in *E. coli* infection [18,20,21].

The intestinal “batches model” used in this study is a static model adapted from the SHIME^®^ (Simulator of Human Intestinal Microbial Ecosystem) and developed to simulate human or animal microbiota fermentation [22,23]. An initial contact test with the intestinal microbiota is performed in a single fermenter, which in this case represents the distal section of the gastrointestinal tract. Weaning is a critical period for piglets and significant compositional and functional differences have been reported in the piglets’ microbiome [24]. This model allowed for a preliminary approach to the microbiota evolution in treated piglets with phage-based treatments.

The aim of this study was to isolate, characterize and assess in vivo, in a *G. mellonella* larva model, and in vitro, in a batches model, the new bacteriophages targeting ETEC.

## 2. Materials and Methods

### 2.1. Bacterial Strains

One *E. coli* of porcine origin belonging to serogroup O8 and fimbriae type F18ab (A-I-210), previously described, was selected for bacteriophage isolation [25]. An *E. coli* serogroup collection of 176 strains, previously characterized by seroagglutination, was used for the host range characterization of these phages [19]. In addition, nineteen strains, isolated from pigs affected by diarrhea and characterized by PCR to confirm their fimbriae type (F4 and F18), were used to determine the lysis activity of the isolated phages [25]. All bacteria were grown in LB Lennox broth (VWR, Leuven, Belgium) at 37 °C.

### 2.2. Phage Isolation

Wastewater samples used for isolation were collected in 2020 in wastewater treatment plants from Liege, Brussels, and one hospital in Ghent (Belgium). Pig manures from the experimental farm of the veterinary faculty of the University of Liege and from a working farm located in Grandrieu (Belgium) were also collected for the isolation of bacteriophages. *E. coli* strain A-I-210 was selected for isolation by enrichment method and propagation. Samples were centrifuged for 10 min at 9500× *g* and filtered at 0.45 μm and then 0.22 μm. The bacterial culture was subsequently grown to obtain an optical density (OD) of 0.3, corresponding to a concentration of 10^8^ CFU (Colony Forming Unit)/mL. The filtered samples were mixed with an equivalent volume of LB Lennox (1 mM CaCl_2_, 1 mM MgSO_4_, VWR, Leuven, Belgium) concentrated twice (LB2X), as well as 150 μL of bacterial culture. A sterility control and a growth control were performed for each sample, the first containing the filtrate and LB2X, and the second containing sterile water, LB2X and 150 μL of bacterial culture.

All tubes were incubated for 6 h with gentle agitation at 37 °C until a putative lysis observation. If present, the lysis tube and the growth control were then centrifuged for 10 min at 9500× *g* and filtered at 0.45 and 0.22 μm. The filtrate (100 μL) from the lysis tube was diluted 10 times in TN buffer (Sigma-Aldrich, Saint-Louis, MO, USA). Drops of 2 μL of the filtrates were spread out on LB Lennox agar (1 mM CaCl_2_, 1 mM MgSO_4_) and covered with a bacterial overlay (OD: 0.3). The plates were incubated at 37 °C overnight or until the appearance of lysis plaques was observed. Three subcultures were then carried out successively from a single plaque. A lysate was obtained by adding 150 μL of the isolated phage and 50 μL of the bacterial culture to 5 mL of LB Lennox. After the observation of lysis in the tube, the lysate was centrifuged, filtered and finally stored at 4 °C.

### 2.3. Phage Titration and Host Range

A 10-fold dilution of the lysate was performed by adding 20 μL of the lysate in 180 μL of TN in 96-well plates. Drops of 2 μL of each dilution were subsequently spotted in triplicate on an LB Lennox agar previously covered with a bacterial overlay. After 3 h of incubation at 37 °C, the lysis plaques were counted and the titer was calculated. For the host range experiment, 4 μL of the lysate of each bacteriophage were deposited in duplicate on LB Lennox agar covered with a bacterial overlay. Plates were incubated at 37 °C for 6 h or overnight. Lysis plaques were then scored (N: no lysis, 1: total lysis, 2: partial lysis, 3: opaque lysis).

### 2.4. Temperature and pH Stability

The effect of temperature on bacteriophage stability was assessed by incubating 1 mL of 10^9^ PFU (Plaques forming Unit)/mL of the phage lysate at different temperatures (25 °C, 37 °C, 45 °C and 60 °C) at pH 7. To evaluate the pH stability, 100 μL of 10^9^ PFU/mL phage lysate was diluted in 900 μL of PBS at different pH (2, 4, 6, 8, 10 and 12). All analyses were performed in triplicate, incubated at 37 °C for 1 h and subsequently titrated with serial dilutions.

### 2.5. Genomic Analysis

DNA extraction was performed by mixing 1 mL of phage solution with 5 μL of DNase I (100 μg/mL) (Sigma-Aldrich, Darmstadt, Germany) and 10 μL of RNase A (100 μg/mL) (Sigma-Aldrich, Darmstadt, Germany) in a sterile tube. After incubation during 1 h at 37 °C, 50 μL of 10% SDS (Merck, Darmstadt, Germany), 5 μL of proteinase K (100 μg/mL) (Sigma-Aldrich, Darmstadt, Germany) and 40 μL of EDTA (0.5 mol/L) (Sigma-Aldrich, Darmstadt, Germany) were successively added. Subsequently, samples were incubated during 1 h at 55 °C. Then, 200 μL of NaCl (5 mol/L) (Sigma-Aldrich, Darmstadt, Germany) and 700 μL of chloroform/isoamyalcohol (24/1, Merck, Darmstadt, Germany) were added. Samples were next incubated at room temperature (RT) for 10 min with frequent inversion and centrifuged at 15,000× *g* for 15 min. The upper phase was next transferred to a new tube. The DNA was precipitated by an adding equal volume of isopropanol (Fisher Scientific, Parsippany, NJ, USA). The tube was inverted several times and then centrifuged at 15,000× *g* for 15 min. The supernatant was removed and the pellet was washed with 1 mL of 70% ethanol (VWR, Leuven, Belgium). After centrifugation at 15,000× *g* for 2 min, the supernatant was finally removed and the pellet was dried for 30 min at RT. The DNA was eluted in 20 μL MilliQ water (VWR, Leuven, Belgium) and stored at −20 °C.

After DNA extraction, sequencing libraries were constructed using an Illumina Nextera XT DNA sample preparation kit, subsequently sequenced using an Illumina MiSeq instrument (Illumina, San Diego, CA, USA) and assembled into single contigs using SPAdes assembler v3.10.0 in the software Geneious v10.2.3. Gene structural and functional annotation was performed through the RAST tool kit (Rapid Annotation using Subsystem Technology) using Patric v3.6.12. Protein functions predicted were then reanalyzed with InterPro v91.0 [26] and HHpred v57c8707149031cc9f8edceba362c71a3762bdbf8 (https://toolkit.tuebingen.mpg.de/, accessed on 2 May 2022) [27,28]. The determination of the lytic or lysogenic lifecycle was predicted using Phage AI. A phylogenetic tree and comparative genomics of phages were constructed using VIPtree v3.5 (https://www.genome.jp/viptree/, accessed on 2 May 2022).

### 2.6. Transmission Electron Microscopy

Transmission electron microscopy (TEM) was performed by the Electron Microscopy Laboratory of the Center for Microscopy and Molecular Imaging (Gosselies, Belgium) on the lysates of filtrated bacteriophages. The grids were placed on a sample drop for 1 min and then on a drop of 4% uranyl acetate for 2 min with paper blotting between each step. The TEM preparations were examined using a Tecnai 100KV microscope (Thermofisher, Waltham, MA, USA).

### 2.7. Galleria Mellonella Larvae Model

Firstly, the optimal inoculation dose was determined by inoculating 7 groups of 10 *G. mellonella* larvae using an automatic injector (Cole Parmer, Vernon Hills, IL, USA) with 10 μL of the A-I-210 strain at 6 different concentrations ranging from 10 CFU/10 μL to 10^6^ CFU/10 μL and with 10 μL of PBS for the last group. Each larva was inoculated in the last left proleg with a BD Plastipak^TM^ 1 mL sterile syringe (Becton-Dickinson, Franklin Lakes, NJ, USA) and a sterile 30-gauge needle (Terumo Corporation, Tokyo, Japan). The optimal inoculation dose was expected to cause a lethality between 90 and 100% after 4 days.

Then, the survival of *G. mellonella* larvae was determined as part of the evaluation of the individual efficacy of vB_EcoS_ULIM2. In total, 150 larvae were required for the experiment and divided into 5 groups (Table 1). Each larva was inoculated using an automatic injector (Cole Parmer, Vernon Hills, IL, USA) in the last left proleg for the first injection followed by a second injection 1 h later in the last right proleg. BD Plastipak^TM^ 1 mL sterile syringes (Becton-Dickinson, Franklin Lakes, NJ, USA) and sterile 30-gauge needles (Terumo Corporation, Tokyo, Japan) were used to perform the injections. Larvae were then incubated at 37 °C.

**Figure 1:**
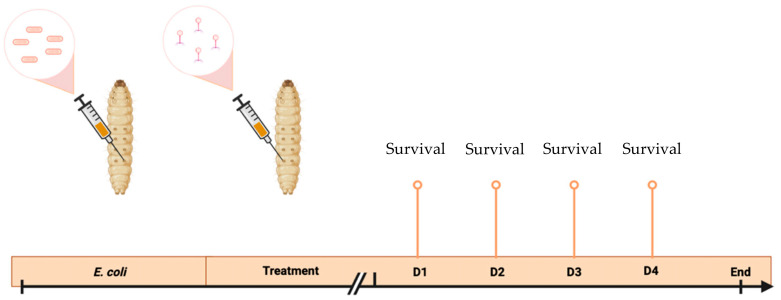
Representation of the *Galleria mellonella* larvae survival experiment. D: Day.

Mortality was evaluated every 24 h for 4 days (Figure 1). Kaplan–Meier survival curves were generated to assess the survival of the different groups using GraphPad Prism 9 (Version 9.4.0 (453)). Logrank tests were performed to highlight any significant differences in survival rates between the groups [19].

### 2.8. Intestinal Batches Model

The intestinal batches model, a static model adapted from the SHIME^®^ model, lasted 72 h, with samples collected at day 0 and every 24 h [29,30,31]. For this experiment, 5 batches simulating the distal part of piglet’s intestine were used.

Fecal samples from 37 piglets aged 24 days were collected at the experimental farm of the veterinary faculty of the University of Liege and transported under cooled and anaerobic conditions. They were pooled and prepared as a 20% (m/m) suspension in phosphate buffer after collection. The suspension was homogenized and filtered using a stomacher bag containing a mesh screen liner (80 μm pore size) (Biomérieux, Basingstoke, UK). Then, 20% of glycerol was added as a cryoprotectant to the filtered fecal material. Fecal samples were kept at −80 °C until inoculation.

Each batch contained 200 mL of a nutritional media representing piglet feed, called Baby L-SHIME growth medium (Prodigest, PD-NM005, Gent, Belgium), and 10 mL of piglet fecal matter. The system was maintained at 39 °C with constant agitation of 300 rpm. Regarding pH, each batch was continuously adjusted between pH 5.9 and 6.1 by automatically adding HCl (0.5 M) or NaOH (0.5 M) (ChemLab, Zedelgem, Belgium). A nitrogen flow (2.0 L/min) was applied daily for 10 min and after each sampling to maintain anaerobic conditions. The first sampling was performed just after adding the feces to the nutrient medium. Then, 10 mL of bacteriophage-based solution (10^8^ PFU/mL) was tested in triplicate while two fermenters were reserved for controls. Samples for metagenetic analysis were centrifuged to recover the pellets and stored at −20 °C until analyses were performed. All these steps were repeated daily for three days to analyze the evolution of the piglet microbiota.

### 2.9. Short-Chain Fatty Acids Analysis

Samples were analyzed for their short-chain fatty acid (SCFA) content. The analyzed compounds were acetic (C2), propionic (C3), isobutyric (iC4), butyric (C4), isovaleric (iC5), valeric (C5) and hexanoic acids (C6). The SCFA analysis was performed following a validated method for SHIME^®^ samples [24]. To a 25 μL batches sample, 40 μL of 0.2 mg/mL methyl-valeric acid in water was added as internal standard, followed by the addition of 15 μL of 0.9 M sulfuric acid and 920 μL of water to obtain 1 mL. The solution was then vortexed and analyzed using solid phase micro extraction (SPME) gas chromatography coupled to mass spectrometry (Thermo Fisher Scientific, Waltham, MA, USA).

### 2.10. Metagenetic Analysis

The metagenetic analysis was performed following the guidelines for 16S metagenomic sequencing library preparation for Illumina technology users. Microbial DNA was extracted from frozen pellets of batches samples with the DNeasy Blood & Tissue kit according to the manufacturer’s instructions (Qiagen Benelux B.V., Venlo, The Netherlands). The V1-V3 regions of 16S rRNA genes were amplified through PCR and then the products were cleaned-up, purified and quantified. All statistical analyses for SCFA concentration and metagenetic analysis data were performed using the software package RStudio [23].

Subsample datasets with 10,000 reads were obtained and used to evaluate alpha diversity (richness estimation—Chao1 estimator, microbial biodiversity—reciprocal Simpson index and Shannon index) using MOTHUR and Benjamini–Hochberg FDR for multi-testing correction. Beta diversity (bacterial community composition) was assessed using the distance matrix based on the Bray–Curtis dissimilarity index. A significant differential population abundance between the pair of groups was identified using Deseq2 package in R. The differences were considered significant at *p* values < 0.05 [32].

## 3. Results

### 3.1. Phage Isolation

Twenty wastewater samples previously and newly collected from different locations were tested to isolate bacteriophages active against one bacterial strain. Four phages were isolated against the A-I-210 ETEC strain. All phages were amplified and titrated to reach approximately 10^8^ PFU/mL.

### 3.2. Host Range

**Figure 2:**
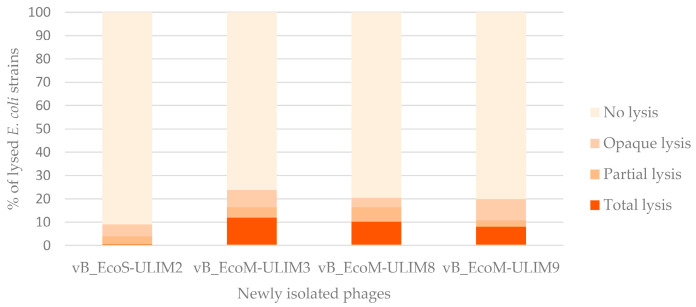
Percentage of the *E. coli* strains belonging to a collection of different serogroups lysed by at least one of the four newly isolated phages.

The host range was performed on the four newly isolated phages, named vB_EcoS_ULIM2, vB_EcoM_ULIM3, vB_EcoM_ULIM8 and vB_EcoM_ULIM9, against the A-I-210 strain. First, these bacteriophages were tested on an *E. coli* serogroup collection containing 176 strains. vB_EcoS_ULIM2 showed the narrowest spectrum (<10% of lysed strains) while the remaining three phages had a wide host range with a percentage of lysed strains ranging from 20 to 24% (Figure 2). A table listing the different lysed serogroups is available in Supplementary File Table S1.

The second part of the host range was evaluated based on the collection of 19 strains of *E. coli* previously characterized according to their type of fimbriae. All bacteriophages were active on several F18 strains, whereas only two phages were able to totally lyse one fimbriae type 4 strain (Table 2).

### 3.3. Temperature and pH Stabilities

**Figure 3:**
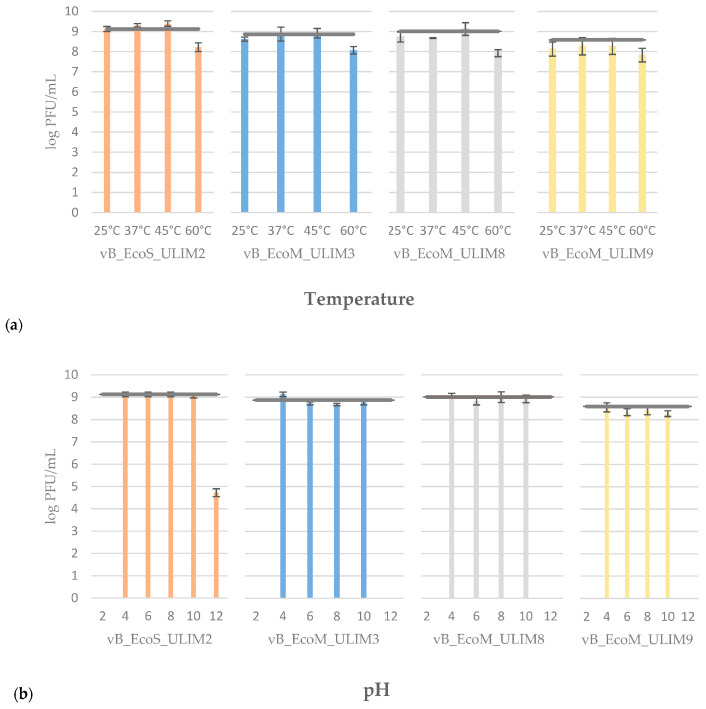
Temperature (**a**) and pH (**b**) stability results of phages vB_EcoS_ULIM2, vB_EcoM_ULIM3, vB_EcoM_ULIM8 and vB_EcoM_ULIM9. The results are the mean value of three titrations. Standard deviations are indicated and the grey horizontal bar represents the titer measured before testing.

All phages were thermostable up to 45 °C (<1 log PFU/mL reduction) and showed a decrease to 8 log PFU/mL at 60 °C (Figure 3a). Concerning the pH stability, the four phages showed stable lytic activity over the pH range of 4–10, but only vB_EcoS_ULIM2 was present at pH 12 with a loss of 4 log PFU/mL (Figure 3b).

### 3.4. Genomic Analysis

**Figure 4:**
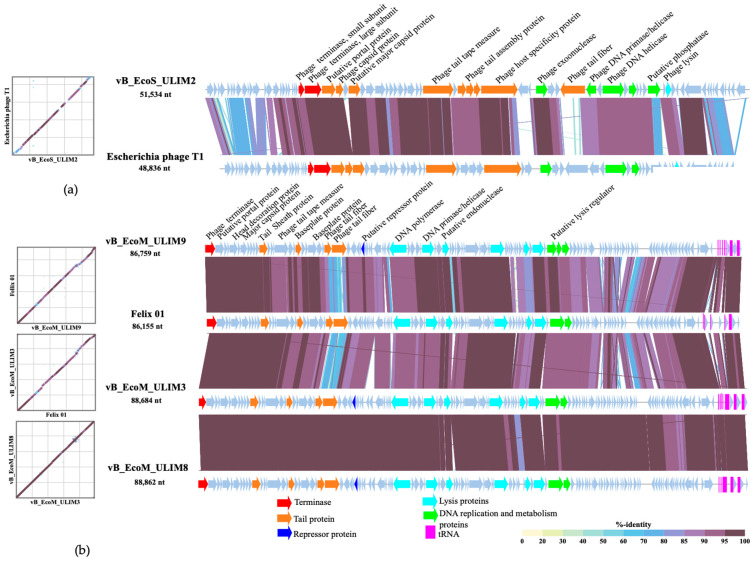
Alignment of the whole genomes of phages vB_EcoM_ULIM3, vB_EcoM_ULIM8, vB_EcoM_ULIM9 with Felix 01 (**a**) and vB_EcoS_ULIM2 with *Escherichia* phage T1 (**b**). The functional categories of the CDS are indicated by specific colors, according to the legend, and the main features are cited above the sequence. Hypothetical proteins are colored in grey. This figure also provides pairwise dot plots of sequences included in the alignment. A color bar on the upper left corner of the view represents %-identity shown in the alignment and dot plots.

Phage genome analysis showed that the four phages have linear double-stranded DNA and belong to the *Caudoviricetes* class. Phage vB_EcoS_ULIM2 in the *Drexlerviridae* family and *Tunavirinae* subfamily has a genome size of 51.534 bp. Phages vB_EcoM_ULIM3, vB_EcoM_ULIM8 and vB_EcoM_ULIM9 are related to the *Felixounavirus* genus in the *Ounavirinae* subfamily with genome size of, respectively, 88.684 bp, 88.862 and 86.759 bp. The clustering in a phylogenetic analysis reflects the number of similar genes and the degree of similarity of those genes in phages of the same family, which was confirmed for vB_EcoM_ULIM3, vB_EcoM_ULIM8 and vB_EcoM_ULIM9 (Figure S1). No gene related to lysogeny or virulence was revealed in vB_EcoS_ULIM2, suggesting the strictly lytic characteristics of this phage. In contrast, the genomes of the three other phages suggested the presence of a questionable protein called P22 C2 repressor. Nevertheless, according to Phage AI S.A. (V. 0.11.0), these four bacteriophages are considered as lytic phages with a percentage of predicted lifecycle of 93.27% for vB_EcoS_ULIM2, 99.30% for vB_EcoM_ULIM3, 98.77% for vB_EcoM_ULIM8 and 98.88% for vB_EcoM_ULIM9. The genomes of vB_EcoM_ULIM3, vB_EcoM_ULIM8 and vB_EcoM_ULIM9 were aligned and displayed with phage Felix01 (AF320576.1), using VIPtree in Figure 4a [33]. Moreover, the alignment of these bacteriophages with JSpecies (v1.2.1.) confirmed their similarity with a percentage of 97.85% between vB_EcoM_ULIM3 and vB_EcoM_ULIM8, 94.32% between vB_EcoM_ULIM3 and vB_EcoM_ULIM9, and finally 94.18% between vB_EcoM_ULIM8 and vB_EcoM_ULIM9. In Figure 4b, vB_EcoS_ULIM2 was aligned with *Escherichia* phage T1 (NC_005833.1). They share a percentage of identity of 94.29% according to Blastn. Supplementary details on Illumina sequencing are reported in Table S2. Sequencing data were submitted as NCBI BioProject PRJNA909979. The Genbank accession numbers are SRR23097344 (vB_EcoS_ULIM2), SRR23097342 (vB_EcoM_ULIM3), SRR23097341 (vB_EcoM_ULIM8) and SRR23097340 (vB_EcoM_ULIM9).

### 3.5. Transmission Electron Microscopy

**Figure 5:**
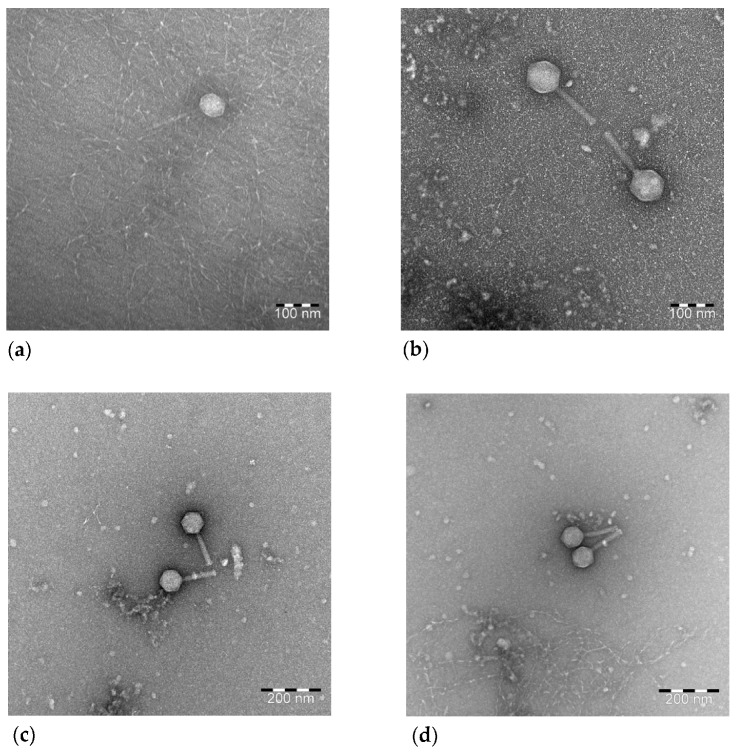
Negative staining transmission electron microscopic images of (**a**) vB_EcoS_ULIM2 with a siphovirus morphology and (**b**) vB_EcoM_ULIM3, (**c**) vB_EcoM_ULIM8 and (**d**) vB_EcoM_ULIM9 with a myovir morphology.

Electron microscopy analyses confirmed that the four phages belong to the *Caudoviricetes* class, according to ICTV Taxonomy. vB_EcoS_ULIM2 was characterized by an icosahedral symmetric non- enveloped head, with a diameter of approximatively 60 nm, and a flexible, non-contractile tail approximatively 135 nm in length, consistent with the siphovirus morphology (Figure 5a). vB_EcoM_ULIM3, with a 90 nm icosahedral head and a 120 nm long contractile tail, was classified as belonging to the myovirus morphology (Figure 5b) along with vB_EcoM_ULIM8 (Figure 5c) and vB_EcoM_ULIM9 (Figure 5d), with, together, icosahedral head diameters of 80 nm and a contractile tail of 100 nm.

### 3.6. Galleria Mellonella Larvae Experiments

**Figure 6:**
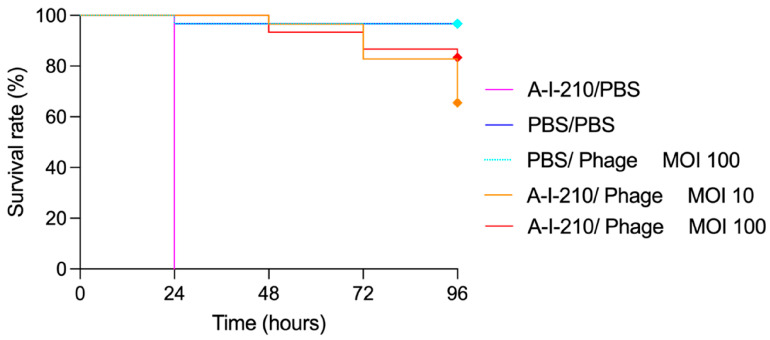
Kaplan–Meier survival curves of *Galleria mellonella* larvae treated with vB_EcoS_ULIM2 at MOI 100 (1 × 10^6^ PFU/mL) and 10 (1 × 10^5^ PFU/mL) 1 h post inoculation with *E. coli* (1 × 10^4^ CFU/mL). *G. mellonella* larvae were monitored at 24 h intervals for 96 h. Each group contained 30 larvae separated into 3 groups of 10 larvae. MOI: multiplicity of infection.

The optimal inoculation dose determined was 10^4^ CFU/10 μL and resulted in the death of all larvae in the infected untreated group 24 h post inoculation. The larvae infected with A-I-210 showed 100% mortality at 24 h and treatment with vB_EcoS_ULIM2 resulted in a statistically significant improvement in the survival rate (*p*-value < 0.001) (Figure 6). The larvae survival rates of the groups treated with vB_EcoS_ULIM2 at a MOI of 10 or 100 were nevertheless not significantly different. vB_EcoS_ULIM2 allowed a better larvae survival rate in the group treated with a MOI of 100 (83% of survival after 96 h). The larvae injected with PBS buffer or phage only (1 × 10^6^ PFU/10 μL) showed less than 5% mortality up to 96 h post inoculation.

### 3.7. Short-Chain Fatty Acid and Metagenetic Analysis

**Figure 7:**
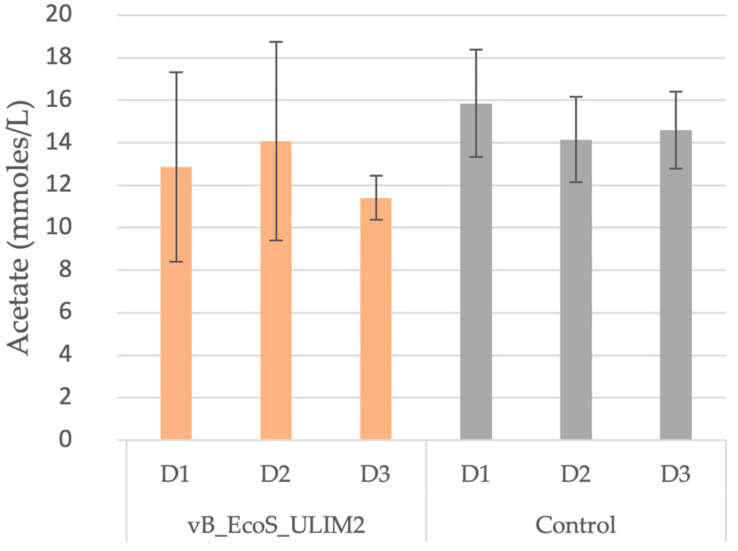
Concentrations of acetate observed in the microbiota in “batches model” at Day 0 and Day 3 post inoculation of vB_EcoS_ULIM2 and a control solution. D: Days.

Regarding the SCFA production, acetate was the only one to be detected and presented, with the other SCFA being non-detectable. For the phage and the control, acetate concentration appeared to remain constant the whole time as no significant changes could be statistically highlighted (Figure 7).

**Figure 8:**
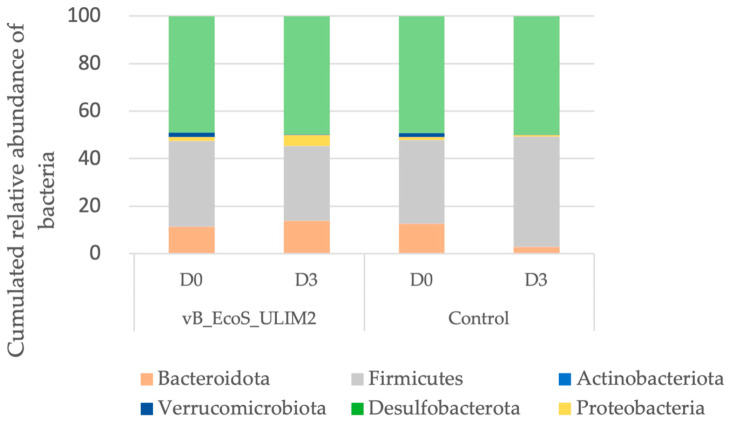
Bacteria composition at phylum level on the microbiota in the batch model at Day 0 and Day 3 post inoculation of vB_EcoS_ULIM2 and a control solution. D: Days.

Regarding the composition of the microbiota overtime, at the phylum level, the *Desulfobacterota* presented the largest relative abundance (50%), followed by *Firmicutes* (from 24% to 47%) and then *Bacteroidota* (from 3% to 21%) (Figure 8). Richness estimation (Chao1 estimator) and microbial biodiversity (reciprocal Simpson index and Shannon index) were used to evaluate alpha diversity with and without inoculation of the phage-based treatment. No significant difference was identified compared to the control (Table 3). The beta diversity using the distance matrix based on the Bray–Curtis dissimilarity index is represented in supplementary file Figure S2.

## 4. Discussion

With the rising mortality and effect on the swine sector, enterotoxigenic *E. coli* is considered as one of the most common pathogens responsible for PWD in piglets [12]. The emergence of antimicrobial resistance has highlighted the need to find other treatments for PWD in pigs [9]. Recently, a resurgence of interest in phage therapy has occurred [34] and positive therapeutic results have been reported several times in animal and human phage therapy [35,36,37].

In this study, several wastewater samples were used to isolate bacteriophages against one F18 ETEC strain. The host range performed for the four newly isolated phages revealed various profiles. Their specificity represents a benefit for the phage contrary to antibiotic treatments, which impact a larger variety of bacterial species and alter the microbiota [19]. The host range also confirmed that isolated phages are much more active against F18 strains, based on several strains characterized according to their fimbriae type.

These four phages presented a narrow spectrum on *E. coli* serogroup collection and a wide range on F4 and F18 strains. It should be mentioned that vB_EcoS_ULIM2 was active against F18 strains but not F4 strains, which could limit its application in veterinary medicine in the context of PWD. Indeed, ETEC F4 appears to be the most prevalent fimbriae type related to PWD, according to research conducted worldwide and particularly in Europe, including historic data from Belgium and France [7]. However, ETEC F18 was shown to have the highest incidence in other countries such as Poland, Cuba, Japan and Spain [38,39].

Phage replication is known to be extremely sensitive to environmental factors [40]. The results of this study supported the ability of these bacteriophages to be stored at different temperatures; however, it may also be of interest to carry out further tests at sub-zero temperatures and at longer time intervals. Indeed, this information is not sufficient for the long-term storage of phages. The administration route of bacteriophage-based therapeutic solutions may depend on pH conditions. Oral administration usually requires the neutralization of the stomach pH or specific formulation to avoid a negative interaction with the treatment [41].

The presence of lysis proteins such as holin and lysin into the phage genomes added to the results of the predicted lifecycle by Phage AI, suggesting that the viruses are lytic phages. This was further confirmed by the fact that all phages lack integrase, which is essential for the lysogenic life cycle [42]. The presence of tRNA genes in the phage genomes is a typical feature [43]. According to Bailly-Bachet et al., the tRNAs are preserved in virulent phage genomes which correlate to codons that are abundant in the phage but uncommon in the host and in phages with a lysogenic cycle [44].

Nevertheless, the putative presence of a repressor-like protein within the genomes of vB_EcoM_ULIM3, vB_EcoM_ULIM8 and vB_EcoM_ULIM9 could raise some questions. In fact, this protein is thought to govern the genetic switch that determines whether an infection will be lytic or lysogenic [45]. Moreover, the roles of the unknown genes such as hypothetical and phage proteins must be further investigated to ensure the safe usage of these phages in therapy.

The bacteriophage was applied at 1 h post bacterial challenge to assess the in vivo phage therapeutic efficacy. In these *G. mellonella* larval tests, larvae treated with vB_EcoS_ULIM2 at a MOI of 10 or 100 had a higher survival rate but not as high as the PBS and phage control groups. The MOI was selected based on previous studies, which demonstrated that an MOI of 10 was the most frequent ratio of phage used against bacterial infections, while an MOI of 0.1 or less resulted in a much lower survival rate [46,47]. In this experiment, the survival rate was significatively higher at an MOI of 100 for vB_EcoS_ULIM2 compared to the infected untreated group (A-I-210/PBS). The test of higher MOIs should be further performed to assess the larvae survival.

The bacterial concentration used (10^4^ CFU/10 μL) resulted in the death of all larvae in the infected untreated group 24 h post inoculation. The improved survival of *G. mellonella* larvae might be due to a quick and temporary drop in bacterial titer during the first 24 h post inoculation. Indeed, research on the *E. coli* O157:H7, bacteriophage Φ241 and the APEC bacteriophage ØEC1 revealed that the host bacteria regrew rapidly after 1 h of infection, resulting in an increase in the bacterial titers [48]. A significant drop in larvae survival occurred 96 h post infection in most phage treated groups. This could be explained by a decrease in the phage titer, which would no longer be able to lyse the bacteria.

A critical phase in the in vitro model of bacterial colonization and steady-state is the stabilization of the various SCFA proportions over time, produced by the polysaccharides fermenting bacteria of the microbiota [49]. Most of the bacterial microbiota community’s energy comes from this source. Additionally, SCFA are beneficial to host health, especially acetate, propionate and butyrate, which induce anti-inflammatory effects and reduce the permeability of the intestinal barrier [50,51]. While many other bacteria known as acetogens are able to produce acetate, *Bacteroides* and *Clostridium* are more specifically known for producing propionate or butyrate [52]. In the context of phage therapy, no significant SCFA production variation was expected over time after treatment [53]. In this study, using a static in vitro model without a stabilization phase, no significant change in the production of acetate was observed after the phage injections. This suggests that the phages do not significantly inhibit bacterial colonization or the model’s homeostasis over time. However, no other SCFA such as butyrate or propionate have been detected, suggesting that this static simplified model presents limitations compared to more complex dynamic models such as the SPIME [24].

The microbiota composition can change over time according to a variety of factors such as age, antibiotic use or diseases [54,55]. Therefore, weaning transition in piglets has been linked to dysbiosis. Although dysbiosis has been described in mammals as a gut microbial imbalance characterized by a marked decrease in the representation of obligate anaerobic bacteria, such as members of the class *Bacteroidia*, and an increased relative abundance of facultative anaerobic bacteria, the characteristics of such a state are not entirely clear [56]. No significant impact could be detected after 72 h of contact of the phage vB_EcoS_ULIM2 with the intestinal piglets’ microbiota, according to ⍺-diversity. However, precaution should be used when interpreting the outcomes in view of the adopted model. In contrast to more specific and dynamic models, the batch model presents limitations, such as relatively short-term perception (48 to 72 h) and parameters that are not entirely adapted to the piglets [24,57]. Moreover, these indexes were estimated using two controls, in addition to a third control, based on the average of the others, which could also constitute a bias. Previous in vivo and in vitro studies have demonstrated the safety of phages against gut microbial compositional changes [53,58,59,60,61]. Nevertheless, in two investigations using mouse models, the authors noted a rise in the ⍺-diversity in conjunction with a hyper-permeability of the gut barrier [62,63]. The interpretation of the metagenomics results may be improved by employing a dynamic model tailored to the piglet, with longer-term experiments and several digestive compartments (ileum, proximal colon).

Phage survival at different time points during the batch model was not determined in this study but would be an interesting piece of information, as another study recently showed that a *Klebsiella pneumoniae* phage gradually disappeared after 7 days in a SHIME system, which suggests that vB_EcoS_ULIM2 could also remain in active concentration during the course of 72 h [64].

In conclusion, this study resulted in the isolation of bacteriophages able to lyse a variety of *E. coli* strains with high specificity or a relatively broad spectrum. One newly isolated phage free of lysogeny related genes was assessed against *E. coli* infection and resulted in a statistically significant improvement in the survival rate in the *Galleria mellonella* larvae model. The safety study of the phage vB_EcoS_ULIM2 in the piglet gut microbiota did not show any significant change in the composition of microbial communities and SCFA production. Further studies are now needed to assess the safety and the efficacy of this phage against ETEC infections in more advanced models.

## Figures and Tables

**Table 1:** Summary table of the *Galleria mellonella* larvae groups inoculated for the survival experiment to assess bacteriophage efficacy.

	Groups	1st Injection (10 μL)	2nd Injection (10 μL)
1	PBS + PBS	PBS	PBS
2	A-I-210 + PBS	A-I-210: 10^4^ CFU	PBS
3	PBS + phage ^1^ MOI 100	PBS	Phage ^1^: 10^6^ PFU
4	A-I-210 + phage ^1^ MOI 10	A-I-210: 10^4^ CFU	Phage ^1^: 10^5^ PFU
5	A-I-210 + phage ^1^ MOI 100	A-I-210: 10^4^ CFU	Phage ^1^: 10^6^ PFU

^1^: vB_EcoS_ULIM2. MOI: Multiplicity of Infection.

**Table 2:** Host range results of four phages against 19 *E. coli* strains characterized by their fimbriae type F4 or F18 (1 = Total lysis, 2 = Partial lysis, 3 = Opaque lysis, N = No lysis).

		Bacteriophages			
*Fimbriae type*	*E. coli* strains	vB_EcoS-ULIM2	vB_EcoM-ULIM3	vB_EcoM-ULIM8	vB_EcoM-ULIM9
**F4**	E65	N	3	3	3
	G7	N	N	N	N
	G205	N	1	1	N
	G491	N	N	3	N
	Abbotstown	N	N	N	N
	24KP88	N	N	N	N
	107KP88	N	N	N	N
**F18**	A-I-9	N	1	1	1
	A-I-85	N	1	1	1
	A-I-136	N	N	N	N
	A-I-137	N	1	1	N
	A-I-154	1	1	1	3
	A-I-164	1	N	N	N
	A-I-210	1	1	1	1
	A-I-219	1	1	1	1
	A-I-220	2	1	1	1
	A-I-222	1	1	1	1
	A-II-37	N	1	3	N
	A-II-40	N	1	1	1

**Table 3:** Average ± SD of Chao1 estimator and Simpson–Shannon index observed in the microbiota in “batches model” at Day 3 post inoculation of vB_EcoS_ULIM2 and a control solution.

	Chao1	Simpson	Shannon
Control	169 ± 81.63	0.49 ± 0.27	1.40 ± 0.64
vB_EcoS_ULIM2	108 ± 33.03	0.25 ± 0.30	2.54 ± 1.18

## Data Availability

Sequencing data were submitted as NCBI BioProject PRJNA909979. GenBank accession numbers for nucleotide sequences: OQ850288, OQ850289, OQ850290, OQ850291.

## Supplementary Materials

The following supporting information can be downloaded at https://www.mdpi.com/article/10.3390/v15051053/s1, Figure S1: Phylogenetic tree representing the relationship between vB_EcoS_ULIM2, vB_EcoM_ULIM3, vB_EcoM_ULIM8 and vB_EcoM_ULIM9, Figure S2: Beta diversity using the distance matrix based on the Bray-Curtis dissimilarity index observed in the microbiota in the batch model 3 days after inoculation of vB_EcoS_ULIM2 and a solution control. Table S1: Effects of isolated phages on a collection of *E. coli* strains organized by serogroups. Table S2: Supplementary data of bacteriophages sequencing by Illumina Nextera XT.
