# Supplementary material for: In Vitro Effect on Piglet Gut Microbiota and In Vivo Assessment of Newly Isolated Bacteriophages against F18 Enterotoxigenic Escherichia coli (ETEC)
Source: Viruses. 2023 Apr 25;15(5):1053. doi: 10.3390/v15051053 (PMC10223428; doi:10.3390/v15051053)
Supplement: Supplementary file 1 [file viruses-15-01053-s001.zip › viruses-2125974-supplementary.pdf]

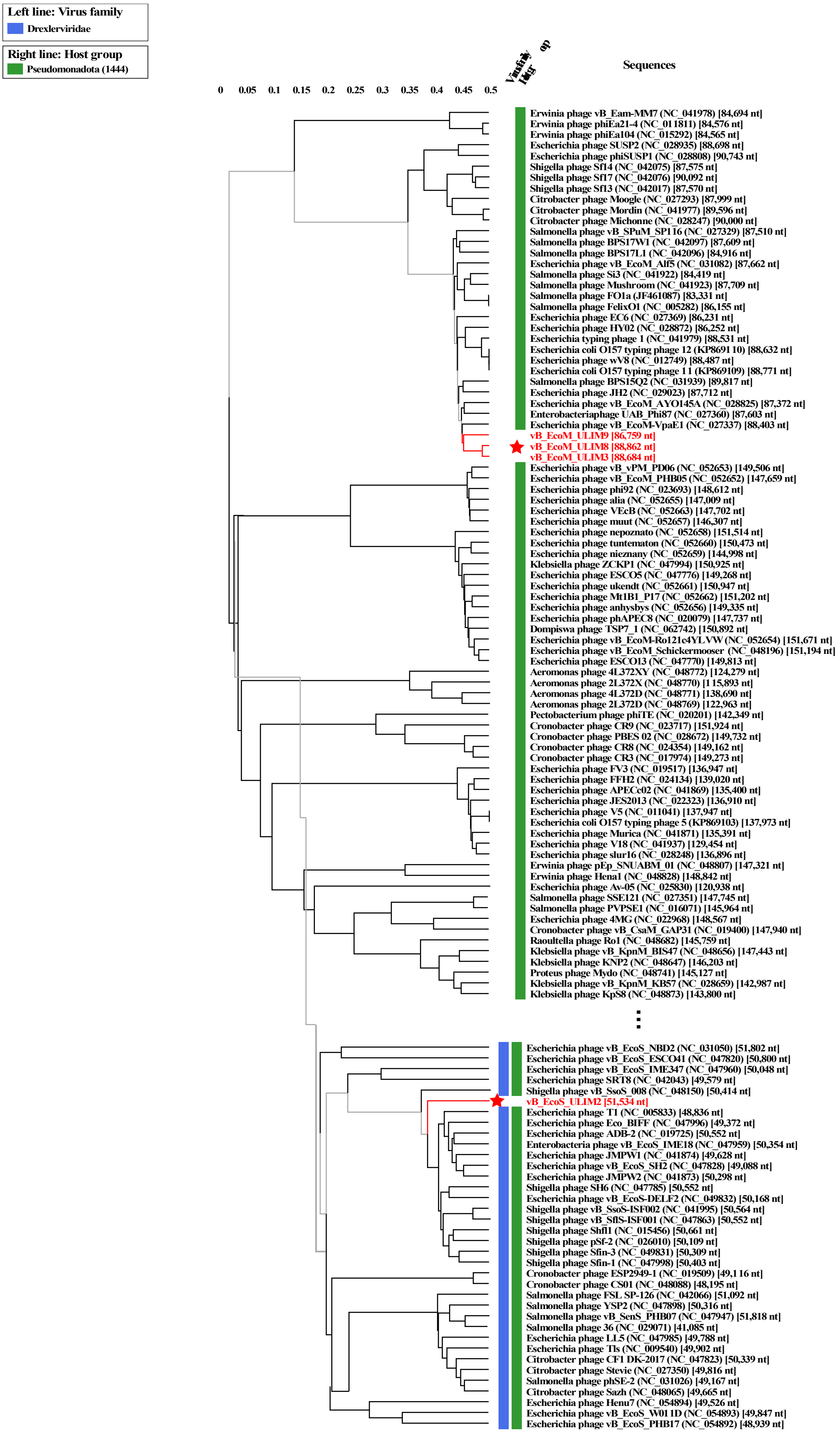


***Figure S1: Phylogenetic tree representing the relationship between vB_EcoS_ULIM2, vB_EcoM_ULIM3, vB_EcoM_ULIM8 and vB_EcoM_ULIM9.***

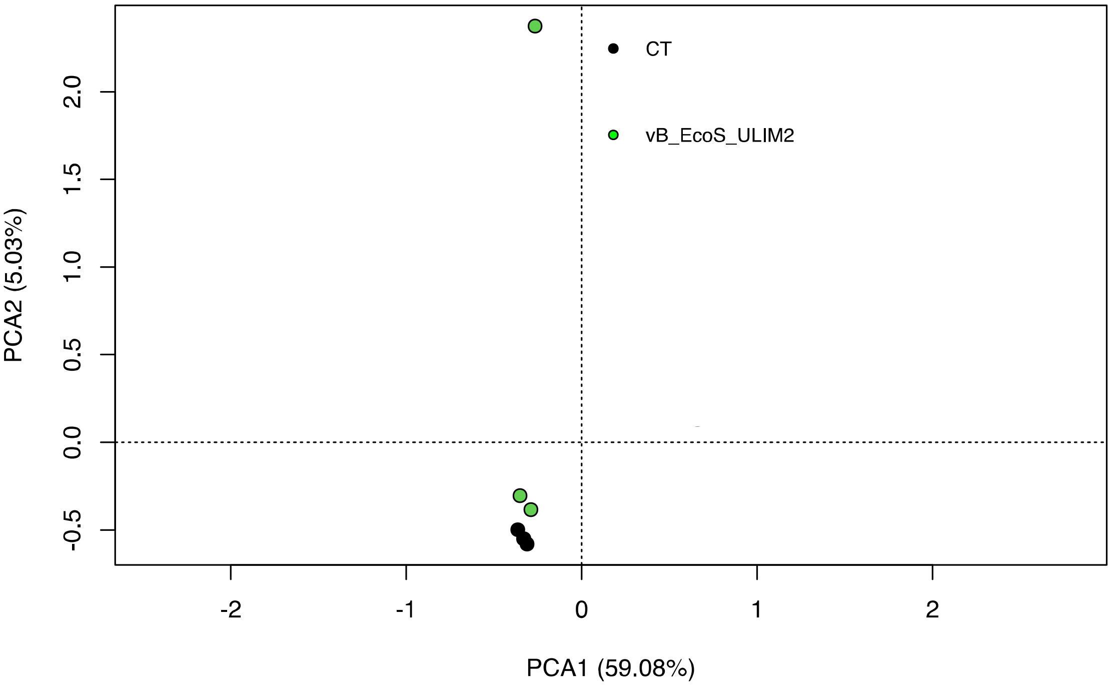


***Figure S2: Beta diversity using the distance matrix based on the Bray-Curtis dissimilarity index observed in the microbiota in the batch model 3 days after inoculation of vB_EcoS_ULIM2 and a solution control.***

*Table S1: Effects of isolated phages on a collection of* E. coli *strains organized by serogroups.*

| Serogroup | vB_EcoS_ULIM2 | vB_EcoM_ULIM3 | vB_EcoM_ULIM8 | vB_EcoM_ULIM9 |
|---|---|---|---|---|
| O1 | + | + | + | + |
| O2 | - | + | - | + |
| O3 | + | + | - | - |
| O4 | + | + | + | + |
| O5 | + | + | + | + |
| O6 | - | + | + | + |
| O7 | + | + | + | + |
| O8 | + | - | - | - |
| O9 | + | + | + | + |
| O10 | + | + | + | + |
| O13 | - | + | - | - |
| O16 | + | + | + | + |
| O18 | - | + | + | + |
| O19 | - | + | - | - |
| O20 | + | + | + | + |
| O22 | - | + | + | + |
| O27 | - | + | + | + |
| O39 | - | + | + | + |
| O40 | - | + | + | + |
| O55 | - | + | + | + |
| O58 | - | + | + | + |
| O64 | - | + | + | - |
| O66 | - | + | + | + |
| O70 | - | + | + | + |
| O80 | - | + | - | - |
| O83 | - | + | + | + |
| O88 | - | + | - | - |
| O93 | + | + | + | + |
| O102 | - | + | + | + |
| 0112ab | + | - | - | - |
| O112ac | + | - | + | - |
| O113 | - | + | + | - |
| O121 | - | + | + | + |
| O124 | - | + | + | + |
| O130 | - | + | + | + |
| O132 | - | + | + | + |
| O136 | - | + | + | + |
| O145 | - | + | + | + |
| O150 | - | + | + | + |
| O152 | - | + | + | + |
| O159 | - | + | + | + |
| O163 | - | + | + | + |

| | | | | |
|---|---|---|---|---|
| O168 | + | - | - | - |
| O169 | + | - | - | - |
| O173 | - | + | + | + |
| O179 | + | - | - | - |
| O181 | - | - | - | + |
| O185 | - | + | + | + |

*Non lysed strains are not represented in the table (+ : Lysis / - : No lysis).*

***Table S2. Supplementary data of bacteriophages sequencing by Illumina Nextera XT.***

| Name | vB_EcoS_ULIM2 | vB_EcoM_ULIM3 | vB_EcoM_ULIM8 | vB_EcoM_ULIM9 |
|---|---|---|---|---|
| **Average read size** | 278.8 | 284.498 | 285.876 | 288.793 |
| **Size of Contig 1** | 51,534bp | 88,684bp | 88,682bp | 86,759bp |
| **Depth** | 365.5x | 226.0x | 184.1x | 184.5x |
